# The impact of operative time on the outcomes of necrotizing soft tissue infections: a multicenter cohort study

**DOI:** 10.1186/s12893-021-01456-0

**Published:** 2022-01-08

**Authors:** Femke Nawijn, Mark van Heijl, Jort Keizer, Paul J. van Koperen, Falco Hietbrink

**Affiliations:** 1 Department of Surgery, University Medical Center Utrecht, Utrecht, The Netherlands; 2 Department of Surgery, Diakonessenhuis, The Netherlands; 3 Department of Surgery, St. Antonius Hospital, Nieuwegein, The Netherlands; 4 Department of Surgery, Meander Medical Center, Hoogland, The Netherlands

**Keywords:** Necrotizing soft tissue infections, Necrotizing fasciitis, Severe necrotizing soft tissue infection, Mortality, Operative time, Damage control

## Abstract

**Background:**

The primary aim of this study was to identify if there is an association between the operative time of the initial debridement for necrotizing soft tissue infections (NSTIs) and the mortality corrected for disease severity.

**Methods:**

A retrospective multicenter study was conducted of all patients with NSTIs undergoing surgical debridement. The primary outcome was the 30-day mortality. The secondary outcomes were days until death, length of intensive care unit (ICU) stay, length of hospital stay, number of surgeries within first 30 days, amputations and days until definitive wound closure.

**Results:**

A total of 160 patients underwent surgery for NSTIs and were eligible for inclusion. Twenty-two patients (14%) died within 30 days and 21 patients (13%) underwent an amputation. The median operative time of the initial debridement was 59 min (IQR 35–90). In a multivariable analyses, corrected for sepsis just prior to the initial surgery, estimated total body surface (TBSA) area affected and the American Society for Anesthesiologists (ASA) classification, a prolonged operative time (per 20 min) was associated with a prolonged ICU (β 1.43, 95% CI 0.46–2.40; *p* = 0.004) and hospital stay (β 3.25, 95% CI 0.23–6.27; *p* = 0.035), but not with 30-day mortality. Operative times were significantly prolonged in case of NSTIs of the trunk (*p* = 0.044), in case of greater estimated TBSA affected (*p* = 0.006) or if frozen sections and/or Gram stains were assessed intra-operatively (*p* < 0.001).

**Conclusions:**

Prolonged initial surgery did not result in a higher mortality rate, possible because of a short duration of surgery in most studied patients. However, a prolonged operative time was associated with a prolonged ICU and hospital stay, regardless of the estimated TBSA affected, presence of sepsis prior to surgery and the ASA classification. As such, keeping operative times as limited as possible might be beneficial for NSTI patients.

**Supplementary Information:**

The online version contains supplementary material available at 10.1186/s12893-021-01456-0.

## Background

Necrotizing soft tissue infections (NSTIs) are potentially lethal infections that cause necrosis of the subcutaneous fat, fascia and/or muscles. NSTIs are notorious for their acute onset and progressive nature, requiring prompt treatment [1]. Bacteria involved in NSTIs can spread rapidly along the fascial planes causing rapid progression and systemic complications [2]. A recent meta-analyses showed that early surgical debridement is vital for lowering mortality rates, since surgery within six hours after presentation lowered the mortality rate for NSTIs with almost 50% [3]. In this review, it was also attempted to identify a relationship between the operative time of the initial surgery for the NSTI and the mortality, the results for this analysis were too scarce. Even though only three studies reported on operative times, these results revealed a possible association between the duration of the initial surgery and the outcome of the NSTI [3–6]. It is well established in trauma and emergency surgery that prolonged operating times potentially lead to higher complication rates [7–9]. Therefore, in critical ill patients with physiological derangement, the damage control surgery principles are more widely applied, specifically in trauma. Damage control procedures are in their initial stage not aimed at definitive repair, but rather aimed to perform limited interventions to control the situation and to recover the patient’s physiology preferable within an operating time of 90–120 min [10, 11]. In trauma patients, this commonly refers to hemorrhage control and prevention of contamination. In NSTI patients this would refer to adequate and rapid source control with temporary closure [10]. After initial surgical control and resuscitation, secondary surgical procedures are required to perform reconstructions and definitive wound closure. However, since information on the association between operative time of the initial debridement for NSTIs and its outcomes is scarce, the aim of this study was to identify if such an association exists and to determine if the principles of damage control surgery are beneficial to NSTI patients.

## Methods

The institutional review board of the initiating hospital provided a waiver (WAG/mb/20/012110) for consent for retrospective data collection. The board of all participating studies approved data collection. A protocol was a priori written, however, not published.

### Study design

A retrospective cohort study of all patients with confirmed NSTIs who underwent their initial surgical debridement at one of the four participating study hospitals (an academic medical center and three large peripheral hospitals) between January 1^st^, 2010 and December 31^st^, 2019 was performed. NSTI refers to the necrotizing forms of fasciitis, myositis and cellulitis. The NSTI had to be confirmed by either operative findings and/or histopathologic tissue findings and/or microbiology results [12, 13]. Patients younger than 18 years at time of onset of the NSTI were excluded, as well were patients who were lost to follow-up after their initial debridement (e.g. due to transfer of patient to another hospital) or if operative times were missing. Eligible patients were identified using different methods per hospital which are outlined in Additional File 1. The study size was based on the number of eligible patients presenting to the study hospitals in the aforementioned study period.

### Data collection

The patient demographics extracted from the medical charts included age, sex, the American Society of Anesthesiologist (ASA) classification. The extracted disease-related characteristics were location of the infection, estimated total body surface area (TBSA) affected, cultured micro-organisms, and laboratory results and hemodynamic parameters just prior to the initial surgery. The TBSA affected was estimated using the rule of nines for burn injuries [14]. Operative times of the initial surgery (incision to end surgery and time in het operating room), (primary or secondary) amputation, if a skin sparing operating technique was used (as reported in the operative note), complications, days until wound closure and mortality (including cause of death and time from initial surgery to death) were among the treatment related variables extracted. If possible, based on the available hemodynamic parameter and laboratory results, the Laboratory Risk Indicator for Necrotizing fasciitis (LRINEC), the sequential organ failure assessment (SOFA) score, the quick sequential organ failure assessment (qSOFA) score and Acute Physiologic Assessment and Chronic Health Evaluation (APACHE) II score were calculated prior to the initial surgery. For the laboratory results and hemodynamic parameters prior to surgery, only values reported within twelve hours prior to the start of the surgery were analyzed. Patients were determined to be septic prior to surgery if either the qSOFA or the SOFA was scored two or higher [15]. The primary outcome of this study was the 30-day mortality. The secondary outcomes were days until death, length of ICU stay, length of hospital stay, number of surgeries within first 30 days, the need for an amputation during re-exploration and days until definitive wound closure.

### Statistical analysis

Normally distributed continuous variables are presented with means and standard deviations (SD), and, if more appropriate based on normality, presented with medians and interquartile ranges (IQR). Categorical variables are presented with frequencies and percentages. Missing data were handled using pairwise deletion. Multivariable analyses (either logistic or linear) were used to determine the association between the operative time per 20 min and the primary and secondary outcomes. For the multivariable analysis, the analysis were corrected for sepsis prior to surgery (imputed as dichotomous variable), ASA classification (imputed as categorical variable) and the TBSA (imputed as continuous variable). These variables were a-priori chosen since the presence of sepsis and the amount and severity of comorbidities influence outcomes are well-known factors associated with outcome such as mortality and length of intensive care and hospital stay [16–19]. The TBSA was chosen since it is very plausible that the area that has to be resected will influence the operative time. The number of covariates chosen to be included in the multivariate analysis was depended on the primary outcome. For the assessment of factors influencing the operative time and mortality, the Mann–Whitney U test was used for dichotomous independent variables, the Kruskal Wallis test for nominal independent variables and the Chi-squared test for trend for ordinal independent variables. For all analyses, a two-sided *p*-value < 0.05 was considered statistically significant. Data will be analyzed using STATA (StataCorp. 2013. Stata Statistical Software: Release 13. College Station, TX: StataCorp LP).

## Results

**Fig. 1:**
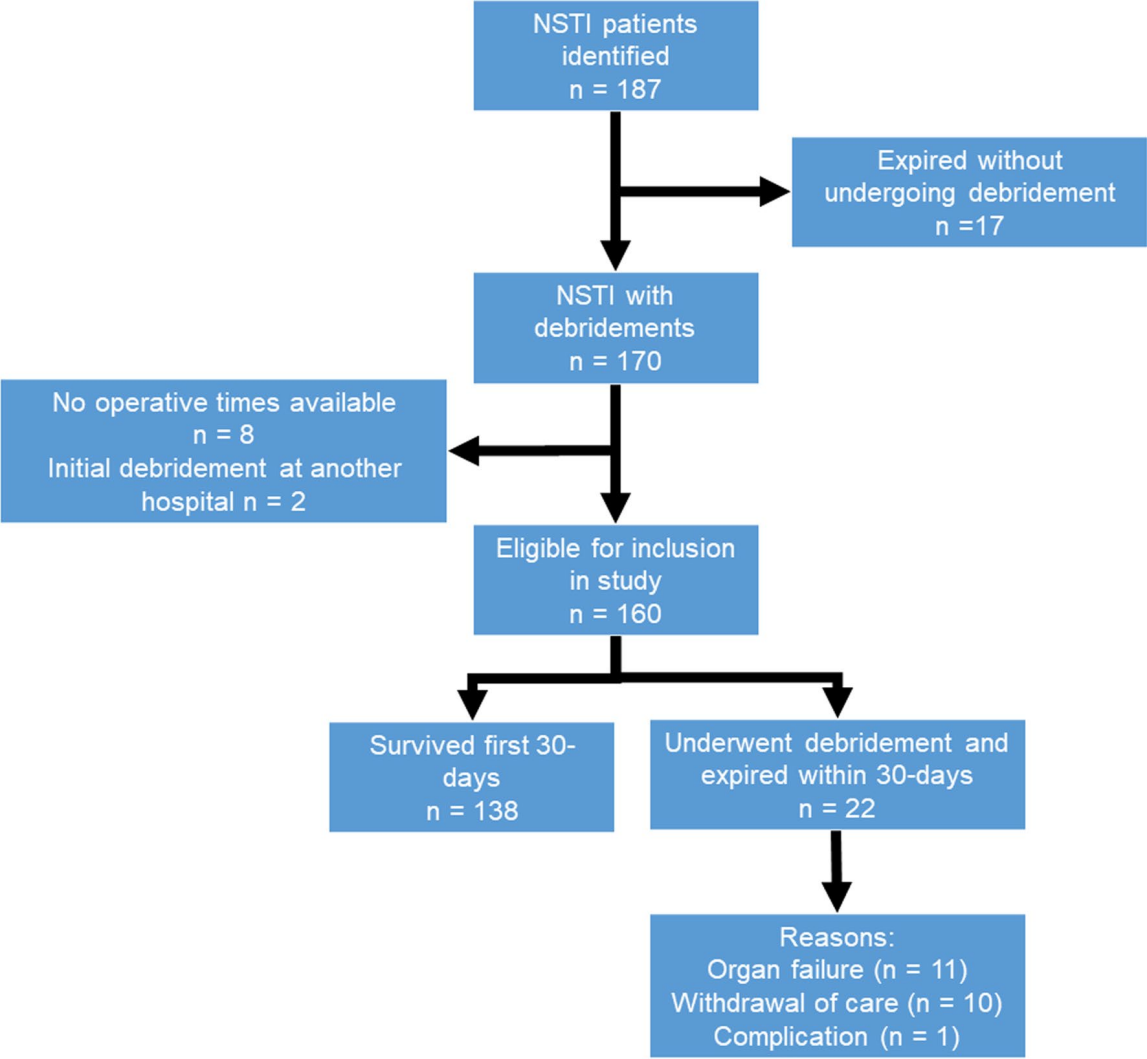
Inclusion flowchart of necrotizing soft tissue infections patients undergoing surgery

A total of 187 patients with NSTIs were identified, of which 160 were eligible for this study (Fig. 1). The mean age of the included patients was 56 ± 16 years. Most patients had no or minor comorbidities (ASA I or II: n = 98, 62%). The lower extremity was most commonly affected by the NSTI (n = 75, 45%). The estimated TBSA affected ranged between 1 and 30% with a median of 3% (IQR 2–6%). Most NSTIs were monomicrobial infection, with group A streptococcus being identified as causative pathogen in almost half of all NSTIs (n = 74, 46%) (Table 1).

**Table 1:** Patient characteristics of necrotizing soft tissue infection patients undergoing surgery

	**n = 160** **(100%)**
Age in years, mean ± SD	56 ± 16
Male sex, n (%)	108 (68)
ASA classification	
I	33 (21)
II	65 (41)
III	52 (32)
IV	10 (6)
Location of NSTI, n (%)	
Head/neck	8 (5)
Trunk	16 (10)
Perineum	44 (27)
Upper extremity	19 (12)
Lower extremity	72 (45)
Multiple body areas involved	1 (1)
Estimated TBSA affected in %^a^, median (IQR)	3 (2–6)
0–5%	111 (70)
6–10%	36 (23)
11–15%	7 (4)
> 15%	4 (3)
Cultured micro-organism from incisional biopsy, n (%)	
Monomicrobial	102 (64)
Group A Streptococcus	74 (72)
Other *Streptococcus spp.*	10 (10)
*Staphylococcus aureus*	7 (7)
*Escherichia coli* *Pseudomonas spp.* *Clostridium spp.* *Other*^*b*^	3 (3) 2 (2) 1 (1) 5 (5)
Polymicrobial	55 (34)
*Escherichia coli involved* *Pseudomonas spp. involved* *Clostridium spp. involved*	18 (32) 3 (5) 7 (12)
Negative cultures	3 (19)

*ASA* American Society of Anesthesiologists; *IQR*  Interquartile Range; *NSTI*  Necrotizing Soft Tissue Infection; *SD*  Standard Deviation; *TBSA* Total Body Surface Area

^a^2 cases missing. ^b^*Acinetobacter baumanii, Enterobacter cloacae*, *Neisseria meningitidis, Proteus vulgaris* and *Vibrio parahaemolyticus*

### Hemodynamic parameters and clinical chemistry prior to surgery

Just prior to the initial surgery, 52 patients (34%) had a systolic blood pressure lower than 100 mmHg, 81 patients (52%) were tachycardic and 40 patients (40%) were tachypnoeic. The mean base excess for all patients was —6.1 ± —5.8. See Table 2 for the mean values of the hemodynamic and laboratory parameters. The median LRINEC score was 7 (IQR 6–9, 22 patients (20%) had a score < 6). Fifty patients (33%) were determined to be septic just prior to their initial surgery for the NSTI, of which 21 (42%) received continuous vasopressors to maintain an adequate mean arterial pressure (Table 2).In Table 3, the factors associated with mortality in patients who were able to undergo surgery for NSTIs are reported. Which shows that within this group of patients mortality is mainly associated with a greater TBSA (*p* = 0.002), higher APACHE II scores (*p* = 0.018) and sepsis prior to surgery (n = 0.047).

**Table 2:** Hemodynamic parameters and laboratory results prior to surgery in necrotizing soft tissue infection patients

	n	Mean ± SD or Median (IQR)	Reference values
Hemodynamic parameters			
Systolic blood pressure	155	114 ± 24	90–120 mmHg
Diastolic blood pressure	155	65 ± 17	60–80 mmHg
Mean arterial pressure	155	81 ± 18	70–100 mmHg
Heart rate	155	104 (88–120)	60–100 beats/minute
Respiratory rate	100	20 (15–25)	12–20 breaths/minute
Temperature	156	37.4 ± 1.1	36–38 °C
Blood test results			
Hemoglobin	94 46	♂ 8.0 ± 1.5 ♀ 7.2 ± 1.2	♂ 8.6–10.7 mmol/L ♀7.4–9.6 mmol/L
Hematocrit	134	37 ± 7	41–50%
Platelet count	118	183 (137–264)	150–450 × 10^9^/L
White blood cell count	146	15.4 (10.2–20.5)	0.8–4.0 × 10^9^/L
Sodium	130	135 ± 6	136–146 mmol/L
Potassium	130	4.0 ± 0.6	3.8–5.0 mmol/L
Creatinine	136	125 (78–184)	64–104 μmol/L
Total bilirubin	82	16 (9–28)	3–21 mmol/L
Lactate	65	3.5 (2.2–5.4)	0.0–2.2 mmol/L
Lactate dehydrogenase	85	235 (194–329)	0–250 U/L
Creatine kinase	61	389 (75–1333)	0–170 U/L
C-reactive protein	146	296 ± 142	0–10 mg/L
Glucose	114	7.0 (5.9–8.4)	3.6–5.6 mmol/L
Arterial blood gas results^a^			
pH	91	7.36 (7.28–7.44)	7.37–7.45
PaO_2_	81	89 (80–132)	70–100 mmHg
PaCO_2_	81	33 (27–40)	35–45 mmHg
Bicarbonate	91	19 ± 5	22.0–28.0 mmol/L
Base excess	91	− 6.1 ± 5.8	− 3.0–3.0 mmol/L
Risk scores			
LRINEC score	108	7 (6–9)	Range 0–13
SOFA score	54	5 ± 3	Range 0–24
APACHE II score Septic upon admission, n (%) Septic prior to surgery, n (%)	55 152 153	13 ± 6 47 (31) 50 (33)	Range 0–67 qSOFA < 2 and/or SOFA score < 2 qSOFA < 2 and/or SOFA score < 2

*APACHE* Acute Physiologic Assessment and Chronic Health Evaluation, *IQR* Interquartile Range, *LRINEC* Laboratory Risk Indicator for Necrotizing fasciitis, *qSOFA* Quick Sequential Organ Failure Assessment, *SD* Standard Deviation, *SOFA* Sequential Organ Failure Assessment

^a^If only a venous blood gas was available, only pH, bicarbonate and base deficit were extracted

**Table 3:** Factors associated with mortality in necrotizing soft tissue infection patients who are able to undergo surgery

	Survived (n = 138, 86%)	Deceased (n = 22, 14%)	*p* value
ASA classification, n (%)			0.057
I–II	89 (91)	9 (9)	
III–IV	49 (79)	13 (21)	
Location of the NSTI, n (%)			
Extremities	78 (86)	13 (14)	0.464
Trunk	13 (81)	3 (19)	1.000
Perineum	39 (89)	5 (11)	0.798
Total body area affected^a^, median (IQR)	3 (2–5)	7 (3–10)	**0.002**
Monomicrobial NSTI^b^, n (%)	86 (84)	16 (16)	0.478
Polymicrobial NSTI^b^, n (%)	49 (89)	6 (11)	0.478
SOFA score prior to surgery^c^, median (IQR)	5 (2–7)	6 (6–9)	0.146
APACHE II score prior to surgery^d^, median (IQR)	11 (9–15)	13 (13–17)	**0.018**
Time from presentation to surgery, median (IQR)	2 (0–3)	1 (0–3)	0.567
Septic prior to surgery, n (%)	39 (78)	11 (22)	**0.047**
Initial operative time^e^, median (IQR)	57 (31–89)	62 (45–90)	0.355

*P*-values in bold denote significant values

*APACHE* Acute Physiologic Assessment and Chronic Health Evaluation, *IQR* Interquartile Range, *LRINEC* Laboratory Risk Indicator for Necrotizing fasciitis, *qSOFA* Quick Sequential Organ Failure Assessment, *SD* Standard Deviation, *SOFA* Sequential Organ Failure Assessment

^a^2 missing cases; ^b^3 missing cases; ^c^106 missing cases; ^d^105 missing cases; ^e^1 missing case

### Impact of operative times on outcomes

**Fig. 2:**
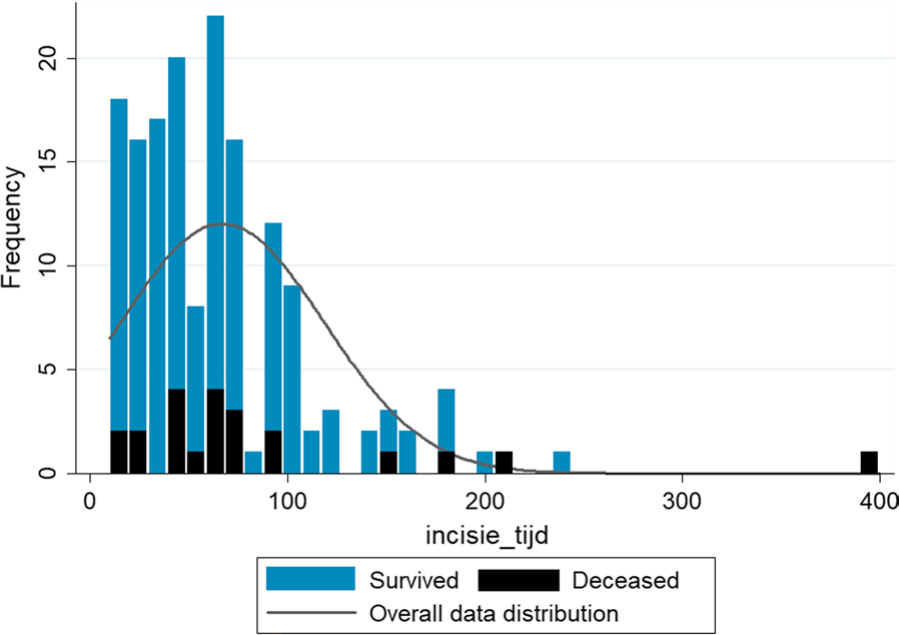
Histogram of distribution of operative times of initial surgery for necrotizing soft tissue infections

The median operative time of the initial debridement for NSTIs was 59 min (IQR 35–90, range 10–400 min), while 35 initial surgeries (22%) took longer than 90 min (Fig. 2). A total of 22 patients (14%) died within 30 days after presentation. The median duration of the initial surgery for deceased patients was 62 min (IQR 45–90) and 57 min for survivors (IQR 31–89, *p* = 0.335). Post-hoc power analysis of our primary objective, the association between the operative time and 30-day mortality, showed a medium effect size (d = 0.30) and a power of 0.28 (α = 0.05, β 0.72). Surgeries that took longer than 140 min had a two-fold higher mortality rate (4/15, 27%) compared to surgeries shorter than 140 min (18/126, 13%), however this differences was not significant with the current sample size (*p* = 0.133).

During 36 surgeries (23%), frozen sections and/or Gram stains were intra-operatively assessed for diagnostic purposes. The use of frozen section and/or gram stain resulted in a shorter time from presentation to diagnosis (4 h (IQR 3–15) vs. 7 h (IQR 4–26), *p* = 0.035), but resulted in significantly longer operative times (*p* < 0.001). In 36 cases (23%) skin sparing operative techniques were utilized, those surgeries had a median operative time of 46 min (IQR 30–90, range 15–400, *p* = 0.715), but was mostly used in cases with relatively low estimated TBSA affected (median 4%, IQR 2–6%). In case of a NSTI affecting the trunk, the initial surgery was significantly prolonged compared to other body locations (*p* = 0.044), this also applied to cases in which a greater estimated TBSA was affected (*p* = 0.006) (Table 4 and 5).

**Table 4:** Treatment characteristics and outcomes of necrotizing soft tissue infection patients undergoing surgery

	**n = 160** **(100%)**
Surgical treatment	
Time from surgical consult to surgery in hours^a^, median (IQR)	7 (4–31)
Operative time of initial surgery in minutes^a^, median (IQR)	59 (35–90)
Time in operating room for initial surgery in minutes^b^, median (IQR)	90 (64–121)
Amputation performed, n (%)	21 (13)^c^
Amputation during initial surgery	13 (62)
Skin sparing operating technique utilized, n (%)	33 (21)
Intra-operative assessed frozen section and/or Gram stain, n (%)	36 (23)
Frozen section, n (%)	22 (14)
Gram stain, n (%)	24 (15)
Number of surgeries for NSTI within first 30 days^d^, median (IQR)	3 (2–5)
Days from initial surgery to definitive wound closure in days^d^, median (IQR)	25 (10–56)
Postoperative phase	
Admitted to ICU, n (%)	110 (67)
Length of ICU stay in days^e^, median (IQR)	4 (2–10)
Length of hospital stay in days^d^, median (IQR)	24 (15–42)
Major infectious complication during hospital course, n (%)	
Sepsis	88 (55)
Multiple organ dysfunction syndrome	14 (9)
Deceased within 30 days after presentation, n (%)	22 (14)
Days from initial surgery to death in days, median (IQR)	2 (1–6)
Cause of death, n (%)	
Sepsis	11 (50)
Withdrawal of care	10 (45)
Complication	1 (5)

*ICU* Intensive Care Unit, *IQR* Interquartile Range, *NSTI* Necrotizing Soft Tissue Infection

^a^1 case missing; ^b^36 cases missing; ^c^Of which 11 were lower extremity amputation, 7 (hemi)scrotectomies, 2 orchidectomies, 1 bilateral mastectomy; ^d^Only patients who survived hospital stay; ^e^Only patients who survived ICU stay

**Table 5:** Factors associated with prolonged operative time for patients with necrotizing soft tissue infections

	Operative time in minutes,	Operative time in minutes,	*p* value
	if yes	if no	
	median (IQR)	median (IQR)	
Location of NSTI			0.158^a^
Head/neck	76 (55–104)	57 (31–90)	0.147
Trunk	90 (59–110)	55 (31–76)	**0.044**
Perineum	49 (36–68)	60 (31–90)	0.474
Upper extremity	45 (19–90)	60 (37–90)	0.368
Lower extremity	59 (35–76)	59 (34–90)	0.650
Estimated TBSA affected			**0.006**^**b**^
< 2%	38 (26–65)	60 (37–90)	0.089
2–3%	45 (22–63)	65 (38–96)	**0.002**
4–5%	72 (38–100)	56 (30–76)	0.134
> 6%	71 (43–100)	47 (29–75)	** < 0.001**
Monomicrobial NSTI infection	59 (30—90)	60 (39—75)	0.711
Amputation during initial surgery	59 (43–93)	59 (35–89)	0.584
Skin sparing operating technique utilized	46 (30–90)	60 (36–89)	0.715
Intra-operative assessed frozen section and/or Gram stain	87 (63–106)	46 (29–73)	** < 0.001**

*P*-values in bold denote significant values

*IQR* Interquartile range, *NSTI* Necrotizing Soft Tissue Infection, *TBSA* Total Body Surface Area

^a^Kruskal Wallis test; ^b^Chi-squared for trend

A multivariable logistic regression, which was corrected for the presence of sepsis just prior to the initial surgery, estimated TBSA and the ASA classification, found no significant association between the operative time and 30-day mortality (β 0.14, 95% CI − 0.06–0.33; *p* = 0.170). There were also no significant associations between the operative time and the need for an amputation during re-exploration (β 0.11, 95% CI − 0.12–0.35; *p* = 0.356), number of surgeries within first 30 days (β 0.01, 95% CI − 0.14–0.16; *p* = 0.873) or days until definitive wound closure (β − 2.77, 95% CI − 7.64–2.09; *p* = 0.262). However, within the linear multivariable analysis, each 20 min increase in operative time resulted in an increase of the ICU stay with 1.4 days (β 1.43, 95% CI 0.46–2.40; *p* = 0.004) and an increase of the hospital stay with 3.3 days (β 3.25, 95% CI 0.23–6.27; *p* = 0.035) (Table 6). The same multivariate analyses, corrected for operative time of the initial surgery, sepsis prior to surgery and ASA classification, showed that an increased estimated TBSA resulted in higher mortality rates (β 0.16, 95% CI 0.04–0.27; *p* = 0.007), longer hospital length of stay (β 6.37, 95% CI 4.19–8.55; *p* < 0.001) and more surgeries within the first 30 days (β 0.25, 95% CI 0.13–0.37; *p* < 0.001). Also sepsis prior to surgery was independently associated with adverse outcomes (corrected for operative time of the initial surgery, estimated TBSA and ASA classification), being an increase in ICU stay with 9 days (β 9.19, 95% CI 4.91–13.47; *p* < 0.001) and the hospital stay with 17 days (β 17.17, 95% CI 3.41–30.94; *p* = 0.015). Septic patients who died (n = 11) did not have a significantly lower operative time compared to septic patients who survived (n = 39) (median of 60 min (IQR 45–76) vs. median 68 min (42–100), *p* = 0.504).

**Table 6:** Association of operative time per 20 min on various outcomes of necrotizing soft tissue infections

	β coefficient (95% CI)	Standard error	*p* value*
Mortality (n = 150)			
Operative time (per 20 min)	0.14 (− 0.06 to 0.33)	0.10	0.170
*Estimated total body surface area affected (in %)*	*0.16 (0.04 to 0.27)*	*0.06*	**0.007**
*Septic prior to surgery*	*0.49 (*− *0.06 to 1.59)*	*0.56*	*0.391*
*ASA classification*	*1.01 (0.31 to 1.72)*	*0.36*	**0.005**
Amputation required after initial surgery (n = 150)			
Operative time (per 20 min)	0.11 (− 0.012 to 0.35)	0.12	0.356
*Estimated total body surface area affected (in %)*	*0.03 (*− *0.13 to 0.20)*	*0.09*	*0.696*
*Septic prior to surgery*	*0.49 (*− *1.04 to 2.02)*	*0.78*	*0.528*
*ASA classification*	*0.26 (*− *0.62 to 1.14)*	*0.45*	*0.568*
Length of ICU stay (n = 104)			
Operative time (per 20 min)	1.43 (0.46 to 2.40)	0.49	**0.004**
*Estimated total body surface area affected (in %)*	*0.29 (*− *0.33 to 0.91)*	*0.31*	*0.352*
*Septic prior to surgery*	*9.19 (4.91 to 13.47)*	*2.16*	**< 0.001**
*ASA classification*	− *1.31 (*− *3.67 to 1.06)*	*1.19*	*0.275*
Length of hospital stay (n = 129)			
Operative time (per 20 min)	3.25 (0.23 to 6.27)	1.53	**0.035**
*Estimated total body surface area affected (in %)*	*6.37 (4.19 to 8.55)*	*1.10*	**< 0.001**
*Septic prior to surgery*	*17.17 (3.41 to 30.94)*	*6.96*	**0.015**
*ASA classification*	*6.84 (*− *0.69 to 14.37)*	*3.80*	*0.074*
Number of surgeries within first 30 days (n = 145)			
Operative time (per 20 min)	0.01 (− 0.14 to 0.16)	0.08	0.873
*Estimated total body surface area affected (in %)*	*0.25 (0.13 to 0.37)*	*0.06*	**< 0.001**
*Septic prior to surgery*	− *0.24 (*− *1.05 to 0.58)*	*0.41*	*0.565*
*ASA classification*	− *0.16 (*− *0.58 to 0.25)*	*0.21*	*0.438*
Days until definitive wound closure (n = 120)			
Operative time (per 20 min)	− 2.77 (− 7.64 to 2.09)	2.46	0.262
*Estimated total body surface area affected (in %)*	− 2.52 (− 6.01 to 0.96)	1.76	0.154
*Septic prior to surgery*	8.32 (− 14.40 to 31.05)	11.47	0.470
*ASA classification*	11.41 (− 1.27 to 24.09)	6.40	**0.077**

Bold values denote significant values

ASA = American Society of Anesthesiologists; ICU = Intensive Care Unit

## Discussion

This study found a median operative time of the initial debridement for NSTIs of 59 min, with most of the debridements (78%) lasting no longer than 90 min and an overall 30-day mortality rate of 14% in NSTI patients who underwent at least one debridement. Greater estimated TBSA affected and a higher ASA classification were independently associated with increased mortality, while the operative time did not demonstrate a direct relation with mortality, however the fifteen patients (9%) who underwent surgery for > 140 min had a two-fold increase in mortality. Multivariate analysis showed that each 20 min of extra operative time during the initial debridement resulted in a 1.4-day increase in ICU stay and 3.3-days increase in hospital stay, even if corrected for the presence of sepsis prior to the surgery, estimated TBSA affected and ASA classification.

No other study has investigated the association between the operative time of the initial debridement for NSTIs and its outcomes. However, three prior studies have reported the mean operative time for their entire NSTI cohort. Hong et al. reported an mortality rate of 60% for fifteen septic *Vibrio* NSTI patient with all a NSTI affecting the extremities, which was associated with a mean duration of the initial debridement of 102 min [5]. Corman et al. found a mortality rate of 4% for Fournier gangrene with an associated mean duration of the initial surgery of 78 min and Elsaket et al. reported an mortality rate of 11.4% for Fournier gangrene associated with a mean duration of the initial debridement of 81 min [4, 6]. Notable, all patients underwent a scrotectomy for source control in the study by Corman et al. and in the study by Elsaket et al. only 5% of the patients were septic upon presentation. As a result, and combined with the fact that those studies only investigated specific NSTI subtypes, these studies cannot directly be compared to our study which consisted of a heterogeneous population with mainly GAS infections in non-Fournier regions as we know that certain pathogens causing NSTIs tend to be associated with higher mortality rates, such as polymicrobial and Vibrio NSTIs [20]. Nonetheless, there is a shorter median operative time in this current cohort, with only 22% of the patients undergoing initial debridements for over 90 min.

As seen in this study, NSTI patients often undergo surgery while they are physiologically compromised (e.g. metabolic acidosis, high sepsis scores), therefore it was postulated that these patients could also benefit from the damage control principles. The concept of damage control was first established to improve outcomes of severely injured trauma patients by obtaining rapid hemorrhage control and prevent contamination without definitive repairs during the first surgery, followed by resuscitation in attempt to prevent and/or reverse the pathophysiological triad of coagulopathy, metabolic acidosis and hypothermia (“lethal triad”) [21]. Definitive surgical repair was reserved until after the goals of resuscitation were reached. In this study reduced operative times were not associated with a reduction in mortality, however, since this study is underpowered for this association, the hypothesis cannot yet be rejected nor confirmed. The lack of mortality reduction might be caused by the fact that the patients included in our study almost all had already a relatively short, and therefore already optimized, operating time. On the other hand, the reduced operative times were indeed associated with a significant shorter ICU and hospital stays, regardless of the presence of sepsis prior to surgery, the estimated TBSA affected and the ASA classification. The concept of reducing length of ICU and hospital stay by reducing operative times has not yet been described for NSTIs, but has been suggested for surgical procedures in trauma and general surgery [8, 22]. Procter et al. studied general surgical procedures and found that the odds ratio for ICU admission, adjusted for operative and patient risk variables, increased with 0.32 each half-hour of extra operative time and the hospital length of stay increased with 6% with each half hour extra operative time [8]. Harvin et al. studied emergency trauma laparotomies and found that damage control principles when applied correctly significantly increased the probability of a shorter ICU and hospital stay compared to when a definitive laparotomies was performed [22]. Furthermore, this study showed that besides operative time, sepsis prior to surgery is also independently associated with prolonged ICU and hospital stay, however this variable is often non-modifiable.

The principle of damage control is based on the philosophy of doing only what is necessary in order not to exhaust the physiological reserves of the patient. Therefore, it can be questioned if performing debridement utilizing the skin sparing technique for NSTIs is doing something more than necessary [23]. In the current study, the skin sparing technique did not result in median prolonged operative times, which might indicate that the technique was used in the proper cases. Nevertheless, a case of 400 min was documented with fatal outcome.

The use of intra-operative diagnostics such as frozen section or Gram stain have also been argued to cause treatment delay, since the time waiting on the results could also be used for debridement, however this statement was not yet investigated in a clinical study [24, 25]. This study found indeed a prolonged operative time with a difference in medians of 41 min, which is to be expected since it can take up to 30 min to process and assess a frozen section [12, 13]. However, the time to diagnosis was significantly shorter in cases that used intra-operative diagnostics (difference in medians of 3 h), which enables timely debridement. However, these intra-operative diagnostic modalities should only be used if indicated: in ambivalent cases to prevent unnecessary debridements in non-NSTI cases or prevent delay and/or refrainment of debridement due to less evident macroscopic findings in NSTI cases [26].

The findings of this study need to be interpreted in context of its limitations. First, the retrospective nature of this study resulted in a substantial amount of missing dating for certain variables, especially limiting our ability to use blood gasses results to calculate SOFA and APACHE II scores and to determine the degree of sepsis/septic shock. Especially for these variables, selection bias is likely present, because patient presenting without systemic toxicity will not always have a comprehensive laboratory work-up. Second, the TBSA was estimated based on operative notes and might be over- or underestimated in certain patients. Third, other factors associated with mortality (such as pathogen or time to surgery) could not reliably be investigated in this study, since only patients undergoing surgery were included and it is known that is common that some patients with NSTIs are already deceased before they can undergo surgery. Furthermore, this study is underpowered regarding the main objective, this warrants further research in bigger cohorts. However, the strengths of this study are the fairly large sample size compared to other NSTI cohorts and that it is the first study assessing the consequences of prolonged operative times of the initial debridement for NSTIs.

## Conclusions

Prolonged initial surgery did not result in a higher mortality rate, possible because of a short duration of surgery in most studied patients. However, a prolonged operative time was associated with a prolonged ICU and hospital stay. The goal remains to prevent treatment delay and to perform an efficient and adequate debridement to obtain source control followed by adequate resuscitation in the ICU. Treating physicians should aim to optimize prompt surgical debridement and start of adequate intravenous antibiotics upon presentation and minimize operative times, since reduced hospital stays will reduce health care costs and has a positive impact on the patient outcomes.

## Supplementary Information

**Additional file 1.** Methods of identifying patients with necrotizing soft tissue infections.

## Data Availability

The dataset used and analysed during the current study are available from the corresponding author on reasonable request.

## Abbreviations

APACHE: Acute Physiologic Assessment and Chronic Health Evaluation
ASA: American Society for Anesthesiologists
CI: Confidence Interval
CK: Creatine Kinase
CRP: C-Reactive Protein
ICD: International Classification of Disease
ICU: Intensive Care Unit
IQR: Interquartile Range
LD: Lactate Dehydrogenase
LRINEC: Laboratory Risk Indicator for Necrotizing fasciitis
MODS: Multiple Organ Disfunction Syndrome
NSTI: Necrotizing Soft Tissue Infection
qSOFA: Quick Sequential Organ Failure Assessment
SD: Standard Deviation
SOFA: Sequential Organ Failure Assessment
STROBE: Strengthening the Reporting of Observational Studies in Epidemiology
TBSA: Total Body Surface Area
WBC: White Blood Cell
