# Supplementary material for: The impact of operative time on the outcomes of necrotizing soft tissue infections: a multicenter cohort study
Source: BMC Surg. 2022 Jan 8;22:3. doi: 10.1186/s12893-021-01456-0 (PMC8742342; doi:10.1186/s12893-021-01456-0)
Supplement: Supplementary file 1 — Additional file 1. Methods of identifying patients with necrotizing soft tissue infections. [file 12893_2021_1456_MOESM1_ESM.docx]

**Additional file 1 Methods of identifying patients with necrotizing soft tissue infections**

| University Medical Center Utrecht | Jan 2010 – Jan 2013 | Patient sought using the International Code for Disease (ICD) 10 for necrotizing fasciitis (M72.6) |
| --- | --- | --- |
|  | Jan 2013 – Dec 2019 | Prospective database of patients with necrotizing soft tissue infection |
| St. Antonius Hospital | Jan 2010 – Sept 2016 | Patients identified using search terms necrotizing fasciitis, Fournier gangrene, myonecrosis in three databases:   - Rare disease list kept by intensive care department - The consulting system of the microbiology department - The microbiology laboratory information management system for documented positive fascia cultures |
|  | Oct 2016 – Dec 2019 | Patient sought using the International Code for Disease (ICD) 10 for necrotizing fasciitis (M72.6) and Fournier gangrene (N49.3) and the Surgical Diagnosis Treatment Combination (DBC) codes for necrotizing fasciitis (164), soft tissue infections (160), large wounds (282) and Fournier gangrene (068 and 098) |
| Diakonessenhuis | Jan 2010 – Dec 2019 | Patient sought using the International Code for Disease (ICD) 10 for necrotizing fasciitis (M72.6) and Fournier gangrene (N49.3) and the Surgical Diagnosis Treatment Combination (DBC) codes for necrotizing fasciitis (164), soft tissue infections (160), large wounds (282) and Fournier gangrene (068 and 098) |
| Meander Medical Center | Jan 2010 – Dec 2019 | Patient sought using the Surgical Diagnosis Treatment Combination (DBC) codes for necrotizing fasciitis (164), soft tissue infections (160), large wounds (282) and Fournier gangrene (068 and 098) |
